# Single-Cell Phenotypic Characterization of Human Pituitary GHomas and Non-Functioning Adenomas Based on Hormone Content and Calcium Responses to Hypothalamic Releasing Hormones

**DOI:** 10.3389/fonc.2015.00124

**Published:** 2015-06-09

**Authors:** Laura Senovilla, Lucía Núñez, José María de Campos, Daniel A. de Luis, Enrique Romero, Javier García-Sancho, Carlos Villalobos

**Affiliations:** ^1^Instituto de Biología y Genética Molecular (IBGM), CSIC, Valladolid, Spain; ^2^Departamento de Bioquímica y Biología Molecular y Fisiología, Universidad de Valladolid, Valladolid, Spain; ^3^Hospital Universitario Del Río Hortega (HURH), Valladolid, Spain; ^4^Departamento de Endocrinología y Nutrición, Hospital Clínico Universitario e Instituto de Endocrinología y Nutrición, Universidad de Valladolid, Valladolid, Spain

**Keywords:** pituitary adenomas, GHomas, somatotropinomas, non-functioning pituitary adenomas, hypothalamic releasing factors, calcium imaging

## Abstract

Human pituitary tumors are generally benign adenomas causing considerable morbidity due to excess hormone secretion, hypopituitarism, and other tumor mass effects. Pituitary tumors are highly heterogeneous and difficult to type, often containing mixed cell phenotypes. We have used calcium imaging followed by multiple immunocytochemistry to type growth hormone secreting (GHomas) and non-functioning pituitary adenomas (NFPAs). Individual cells were typed for stored hormones and calcium responses to classic hypothalamic releasing hormones (HRHs). We found that GHomas contained growth hormone cells either lacking responses to HRHs or responding to all four HRHs. However, most GHoma cells were polyhormonal cells responsive to both thyrotropin-releasing hormone (TRH) and GH-releasing hormone. NFPAs were also highly heterogeneous. Some of them contained ACTH cells lacking responses to HRHs or polyhormonal gonadotropes responsive to LHRH and TRH. However, most NFPAs were made of cells storing no hormone and responded only to TRH. These results may provide new insights on the ontogeny of GHomas and NFPAs.

## Introduction

Pituitary adenomas are non-metastasizing neoplasms of adenohypophyseal cells occurring with an incidence extrapolated from autopsies larger than 20%. In addition, common mutations of oncogenes and tumor suppressor genes present in non-endocrine neoplasms, such as PKC, RAS, P53 and RB, are usually absent in pituitary adenomas. Multiple evidence indicate that pituitary tumors are monoclonal in nature (1) suggesting five well-defined types of pituitary adenomas (1, 2). Pituitary tumors are classically typed according to morphometric and secretory characteristics (3). For example, pituitary tumors secreting growth hormone (GH), called also GHomas or somatotropinomas, represent about 15% of all pituitary tumors detected (4). They are characterized by excess of GH secretion causing acromegaly in adults and gigantism in children. Other frequent pituitary tumors are the non-functioning pituitary adenomas (NFPAs) representing around 20% of all pituitary adenomas. These mostly benign adenomas include null cell adenomas, gonadotroph adenomas staining but not releasing gonadotropins, and silent adenomas resembling silent corticogonadotroph adenomas (5). These patients suffer of tumor mass effects including headache, visual disturbances, and hypopituitarism and are usually diagnosed as macroadenomas after magnetic resonance imaging (MRI).

According to the monoclonal hypothesis, cells from GH-secreting tumors should express functional receptors for GH-releasing hormone (GHRH) and store and release GH. However, paradoxical secretion of alternative anterior pituitary (AP) hormones has been reported in multiple instances of pituitary adenoma, including GHoma patients responding to thyrotropin-releasing hormone (TRH) with GH secretion (6–9) and acromegaly patients producing prolactin (PRL) upon GHRH administration (10). Paradoxical secretion has been also noticed in normal pituitary glands, both *in vitro* and *in vivo* (11, 12). These data suggest that pituitary tumors may comprise cells expressing multiple AP hormones. Consistently, AP cells co-storing thyroid-stimulating hormone (TSH) and GH or PRL (13) have been reported. Multihormonal tumor cells producing GH and TSH have been isolated from patients affected by acromegaly and hyperthyroidism (14) and cells co-secreting GH and PRL have been described in pituitary adenomas from acromegalics (15). Additionally, mRNA for both GH and PRL has been detected in the tumors of patients with a clinical diagnosis of acromegaly or gigantism (16).

We have previously shown that a series of human pituitary adenomas including prolactinomas, pituitary adenomas associated to multiple endocrine neoplasia type I (MEN I) disease, pituitary tumors linked to Cushing’s disease, and a few NFPA contained indeed cells storing more than one AP hormone (multihormonal cells) and/or showing responses to more than one hypothalamic releasing hormone (HRH) (multiresponsive cells) (17). For this end, we used fluorescence imaging of individual cells loaded with fura2 to record the rises in cytosolic Ca^2+^ concentration ([Ca^2+^]_cyt_) induced by specific HRHs added and removed sequentially. It is well established that stimulation of each of the four classic HRH receptors induced a rise in [Ca^2+^]_cyt_, in specific cell subpopulations, thus providing functional evidence of expression of specific HRH receptors in individual cells. Combination of this approach to multiple immunocytochemistry in the very same cells used for calcium imaging provides the advantage of characterizing the hormones stored and the functional HRH receptors expressed at the single-cell level (17, 18).

However, the number of NFPA studied in our former study was very too low and no data were obtained from GHomas. Accordingly, we aimed here at characterizing the phenotype of individual cells from human GHomas and additional NFPAs according to the hormones stored and functional (calcium) responses to the four classic HRHs including TRH, GHRH, corticotrophin-releasing hormone (CRH), and gonadotropin-releasing hormone. For this end, we used a combination of multiple immunofluorescence and calcium imaging applied on the same individual cells reported previously (17, 18).

## Materials and Methods

### Materials

Antisera against human AP hormones FSHβ (AFP 891891), GH (AFPC11981A), LHβ (AFP55951889), PRL (AFP55781789), TSHβ (AFP55741789), and ACTH (AFP39032082Rb) were generous gifts from the National Hormone and Pituitary Program (Torrance, CA, USA) and Dr. A. F. Parlow. Human HRHs (GHRH, TRH, LHRH, and CRH) were purchased from Sigma (Madrid, Spain). Fluorescent antibodies were prepared by labeling with Oregon Green 488, Cascade Yellow, or Alexa 350 and purified over a protein A-Sepharose column (17). Fura-2/AM, Oregon Green 488-isothiocyanate, Cascade Yellow succinimidyl ester, and Alexa 350 succinimidyl ester were purchased from Molecular Probes (Eugene, OR, USA).

### Human pituitary tumor cell culture

All the procedures used here were approved by the Valladolid University Hospital and the Valladolid University School of Medicine ethical committees. Selected patients were asked to sign an informed consent form. Fresh pituitary tumor tissue was obtained at the time of surgery and quickly carried to the laboratory in cold minimal essential medium (MEM, Invitrogen, Carlsbad, CA, USA). Extreme care was taken to ensure that samples used for cell culture were devoid of any contaminating normal tissue. Tissue was transferred to fresh MEM medium at room temperature, washed extensively with the same medium and quickly dispersed with trypsin (1 mg/ml) for 15–30 min at 37°C. Dispersed cells washed twice, plated onto coverslips previously coated with 0.01 mg/ml poly-l-lysine and cultured in Dulbecco’s modified Eagle’s Medium (DMEM, Invitrogen) supplemented with 10% fetal bovine serum and antibiotics for at least 2 h for recovery. Further details have been reported previously (17, 18).

### Calcium imaging

Calcium imaging was carried out as previously reported (18, 19). Briefly, after a few hours of dispersion to allow recovery from trypsin digestion, cells were incubated with Fura-2/AM (4 μM) for about 1 h at room temperature (25°C) in standard medium containing 145 mM NaCl, 5 mM KCl, 1 mM MgCl_2_, 1 mM CaCl_2_, 10 mM Hepes (pH 7.4), and 10 mM glucose. Then, attached cells were washed in the same medium, placed in a thermostatically controlled (37°C) stage of an inverted microscope (Diaphot; Nikon, Tokyo, Japan), and perfused with prewarmed standard medium. Cells were epi-illuminated alternately at 340 and 380 nm, and light emitted above 520 nm was recorded using a Magical Image Processor (Applied Imaging, Newcastle, UK). Pixel-by-pixel ratios of consecutive frames were obtained and cytosolic free calcium concentration ([Ca^2+^]_i_) was estimated from these ratios by comparison with Fura-2/AM standards. Test solutions made of standard medium containing 10 nM HRHs were perfused for 30 s at the times indicated. A depolarizing solution containing 150 mM K^+^ (added in exchange for Na^+^) was perfused for 10 s at the end of the experiment to reveal healthy cells bearing functional voltage-operated Ca^2+^ channels. Cells not responding to the high-K^+^ concentration stimulus (usually <5% of the total) were excluded from the analysis.

### Multiple sequential immunocytochemistry

After calcium imaging, the very same studied cells were typed by the hormone/s they stored using the sequential immunocytochemistry protocol previously reported (17). Briefly, cells on the microscope’s stage were carefully fixed with 4% paraformaldehyde (PFA) in PBS without losing the microscopic field. Then, cells were treated with 0.3% Triton X-100, and washed with PBS in the same conditions. After a few minutes, 10% goat serum in PBS was added. After 5 min, cells were incubated with antibodies against three human AP hormones (TSH, FSH, and LH) labeled with Oregon Green 488, Cascade Yellow, and Alexa 350, respectively, for 30 min. After extensive washing using the perfusion system, fluorescence images corresponding to each fluorophore were taken [Oregon Green (FSH): excitation, 490 nm; emission, >510 nm; Cascade Yellow (TSH): excitation, 380 nm; emission, >510 nm; and Alexa 350 (LH): excitation, 340 nm; emission, >450 nm]. This step enables typing cells storing TSH, LH, or FSH as well as cells co-storing combinations of these hormones. After imaging, cells were again washed extensively using the perfusion system and incubated with a second series of antibodies against GH, PRL, and ACTH labeled with Oregon Green 488 (PRL), Cascade Yellow (GH), and Alexa 350 (ACTH) for 30 min. Then, cells were washed, and three new fluorescence images were taken with the same fluorescence settings described above. This new image series revealed both cells that were stained by the first antibody and those labeled by the second one. Cells stained only by the second series of antibodies were identified by image subtraction. Finally, nuclei were stained with Hoechst 33258 (0.5 μg/ml, 10 min) before a last image was acquired (excitation, 340 nm; emission, >420 nm). Further details and controls for this procedure have been reported previously (17, 18).

## Results

### Strategy for typing of human pituitary adenoma cells

The most relevant clinical and pathological features of 9 human pituitary GHomas adenomas and 11 NFPAs used in this study are summarized in Table 1. Male and female patients were 45–66 years old and selected because they were clinically diagnosed of either acromegaly or NFPA before being subjected to neurosurgery for adenoma ablation. In the case of GHomas, three adenomas out of five that had been studied by immunohistochemistry at the pathology department stained positive for GH and another hormone, supporting the status of “paradoxically secreting tumors.” Paradoxical secretion was suspected, for instance, in patient #4, who presented increased serum levels of PRL. Experiments were conducted to study functional responses to the four classic HRHs and multiple sequential immunocytochemistry (MSPI) for testing single or multiple AP hormone content in the same, individual cells.

**Table 1:** **Clinical features of the human pituitary adenomas used**.

No.	Sex, age	Clinical features	Size (mm)	ICC
1	M, 60	Sexual dysfunction (92 nmol/l IGF-1)	20	GH++
				FSH++
				LH++
2	M, 51	Acromegaly	35	Unknown
2recid	52
3	W, 45	Acromegaly (98 nmol/l IGF-1, 40 ng/ml GH)	10	GH++
4	M, 51	Acromegaly (83 nmol/l IGF-1, 20 ng/ml GH, 183 mg/ml PRL)	15	GH++
				PRL++
5	W, 66	Acromegaly (84 nmol/l IGF-1)	5	GH+++
6	W, 65	Acromegaly	30	Unknown
7	M, 28	Acromegaly	20	Unknown
8	W, 52	Acromegaly (84 nmol/l IGF-1)	Invasive	GH++
				PRL+
				LH+
9	M, 45	Non-functioning, visual defects, headache, low TSH, LH, and testosterone	35	ACTH++
10	W, 55	Non-functioning, visual defects, low TSH, and LH	40	FSH+++
11	M, 76	Non-functioning, hypopituitarism (8 years)	30	ACTH+
12	W, 37	Non-functioning, hyperprolactinemia (40 ng/ml PRL), amenorrhea, galactorrhea	12	Negative
13	M, 59	Non-functioning, hypopituitarism	23	FSH+
14	W, 53	Non-functioning, visual defects	29	ACTH+
15	M, 39	Non-functioning, visual defects, headache, cortisol 8 a.m. 24 g/dl, ACTH a.m. 63 pg/ml	30	ACTH++
16	W, 75	Non-functioning, low thyroid hormones	35	Unknown
17	W, 57	Non-functioning, visual defects, low GH, and LH, ACTH a.m. 80 pg/ml	40	Unknown
18	M, 67	Non-functioning, visual defects, hypopituitarism	30	Unknown
19	W, 60	Non-functioning, visual defects, hypopituitarism	30	LH++
				FSH+
				PRL+

*W, woman; M, man; ICC, Immunocytochemistry*.

*The size of the tumor represents the maximum diameter of the tumor. All the patients with nonfunctioning pituitary adenoma were diagnosed on the basis of visual defects and magnetic resonance imaging*.

**Figure 1:**
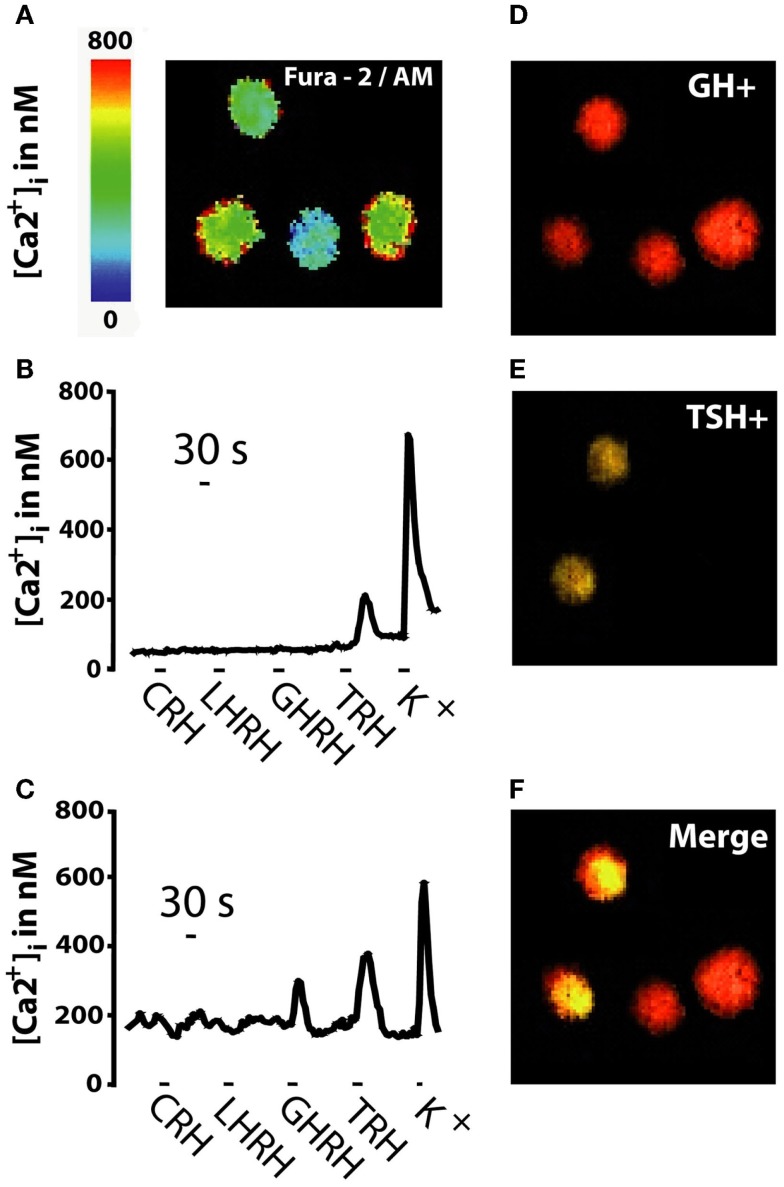
**Strategy for typing of human pituitary adenoma cells**. Pituitary tumor cells were loaded with fura-2 and subjected to Ca^2+^ imaging before sequential immunocytochemistry against the six AP hormones. **(A)** Fura2 image shows calcium levels, coded in pseudocolor (0–800 nM, scale at left) during stimulation with 10 nM GHRH. **(B,C)** Typical [Ca^2+^]_i_ recordings for two individual cells during sequential perfusion with the four HRHs (20 nM) and high-K^+^ medium. Cells with [Ca^2+^]_i_ responses larger than 50 nM were considered responsive. Cells lacking responses to high-K^+^ were excluded from analysis. **(D–F)** Multiple immunocytochemistry images of the same calcium field. Cells storing GH (GH+) are shown in red **(D)**, cells storing TSH (TSH+) are shown in yellow **(E)** and merge image **(F)** shows two monohormonal GH− cells (in red) and two multihormonal GH+/TSH+ cells (in yellow). Data are representative of 547 cells studied in 21 independent experiments.

Figure 1 summarizes the strategy followed for the phenotypic characterization of pituitary adenoma cells, which was established first in mouse AP cells (18) and then in other human pituitary tumors (17). Freshly dispersed pituitary tumor cells were cultured for a few hours and then loaded with fura-2/AM and subjected to calcium imaging to monitor Ca^2+^ responses induced by the four classic HRHs perfused sequentially (CRH, LHRH, TRH, and GHRH) (Figures 1A–C). Increases in [Ca^2+^]_i_ in responsive cells reflects most likely expression of specific, functional HRH receptors (20–24) (Figure 1A). Responsive cells were considered those cells showing an increase in [Ca^2+^]cyt larger than 50 nM either to only a particular HRH (monoresponsive cells, Figure 1B) or to more than one HRH (multiresponsive cells, Figure 1C). At the end of the imaging experiment, cells in the same microscopic field were fixed and subjected to multiple, sequential immunocytochemistry (Figures 1D–F) against the five AP hormones to reveal content of either single or multiple AP hormones in the same cells. Based on this analysis, cells were typed as silent, monohormonal, and multihormonal depending on whether cells stored no apparent hormone, a single hormone, and several AP hormones, respectively. For instance, cells from the same tumor could stain positively for GH only of for both GH and TSH (Figure 1F).

### Characterization of human GHomas

We have used the above strategy to characterize in the first place cells derived from nine human GHomas. Most GHomas displayed distinct phenotypic characteristics, often even among cells from the same tumor. According to hormone storage and calcium responses to the four HRHs, GHomas studied here have been pooled into three different groups according to the results: Type I, non-responsive GHomas; Type II, multiresponsive GHomas, and Type III, TRH and GHRH-responsive GHomas.

**Figure 2:**
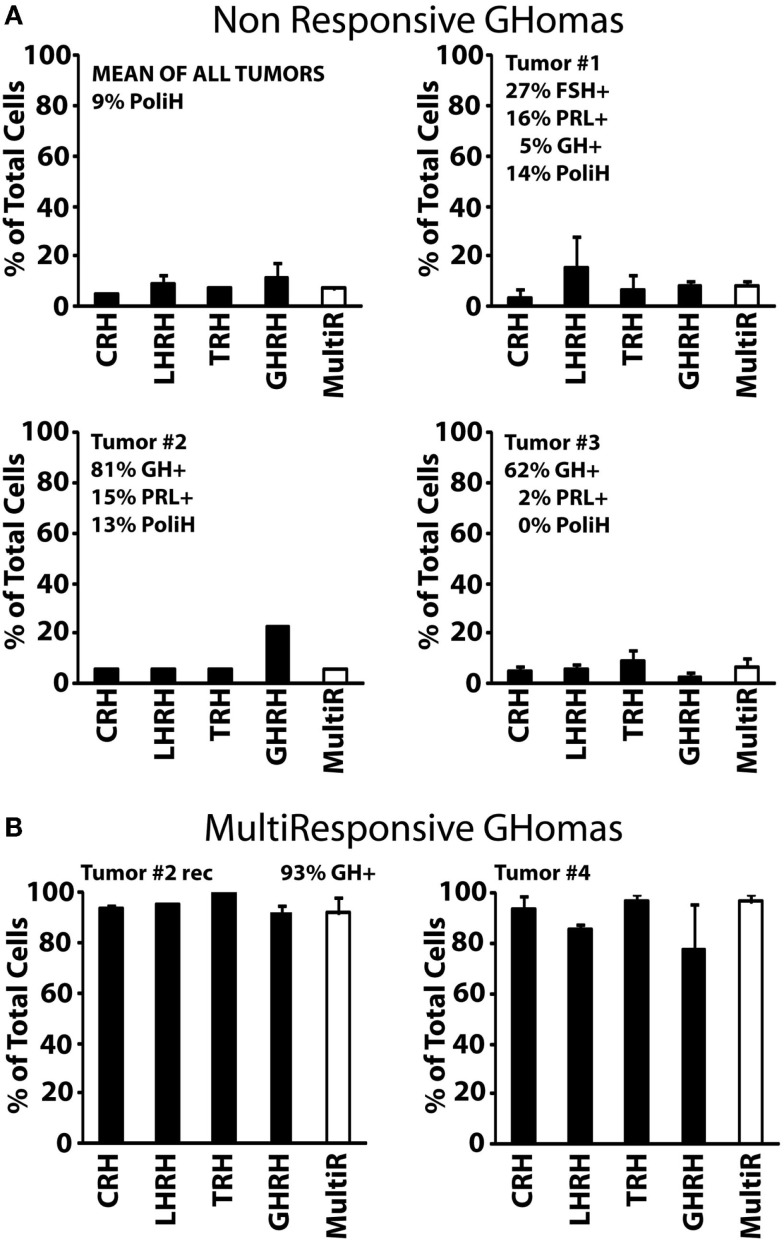
**Phenotypic characterization of non-responsive and multiresponsive GHomas**. **(A)** The analyses of the three non-responsive tumors and their average (up graphic on the left) are shown. The percentage of cells responding to each HRH (with a calcium rise larger than 50 nM) are shown (mean ± SEM). The white bar at right shows the percentage of multiresponsive cells (those showing responses to >1 HRH). The hormonal content of the cells is given in upper right corner of each panel. Data derived from 67 cells (2 experiments, tumor #1), 31 cells (one experiment, tumor #2), and 198 cells (4 experiments, tumor #3). Average data derived from 296 cells studied in 7 independent experiments. **(B)** Phenotypic characterization multiresponsive GHomas. Data from one tumor and one recurrence (Tumor #2rec and #4) are shown. Data derived from 93 cells (two experiments, #2) and 25 cells (two experiments, tumor #4). Clinical and pathological data of these tumors are summarized in Table 1.

Type I (non-responsive GHomas, tumors #1, #2, and #3) includes tumors in which cells lacked generally responses to any HRH or responses were observed only in a minor fraction of cells. In fact, <10% of cells from these tumors responded to CRH, LHRH, TRH, and GHRH, with little or no intra-tumor differences (Figure 2A). The sequential immunocytochemical characterization in the same cells revealed some heterogeneity in terms of hormone content. Whereas tumors #2 and #3 contained mostly GH cells and much less frequently PRL, tumor #1 cells stored no hormone, although a minor fraction of cells stored FSH, PRL, and GH (Figure 2A).

Type II, multiresponsive GHomas included two tumors only, tumor recurrence #2rec and tumor #4. In striking contrast with type I GHomas, nearly all individual cells in these tumors responded to all four HRHs (Figure 2B). Although there are no immunocytochemical results for tumor #4, the patient exhibited an excessive secretion of both GH and PRL. Interestingly, the phenotype of the tumor #2rec differed markedly from the adenoma obtained 1 year earlier from the same patient (tumor #2). Most cells stored indeed GH (93%) as did cells from the primary tumor mass (81%). However, cells from the relapsing tumor (tumor #2rec) responded to HRHs much more extensively that did their primary counterparts, with more than 90% of cells responding to all tested HRHs. Therefore, the relapsing tumor studied showed a marked phenotypic contrast with the original adenoma from the same patient obtained 1 year earlier (Figure 2A).

**Figure 3:**
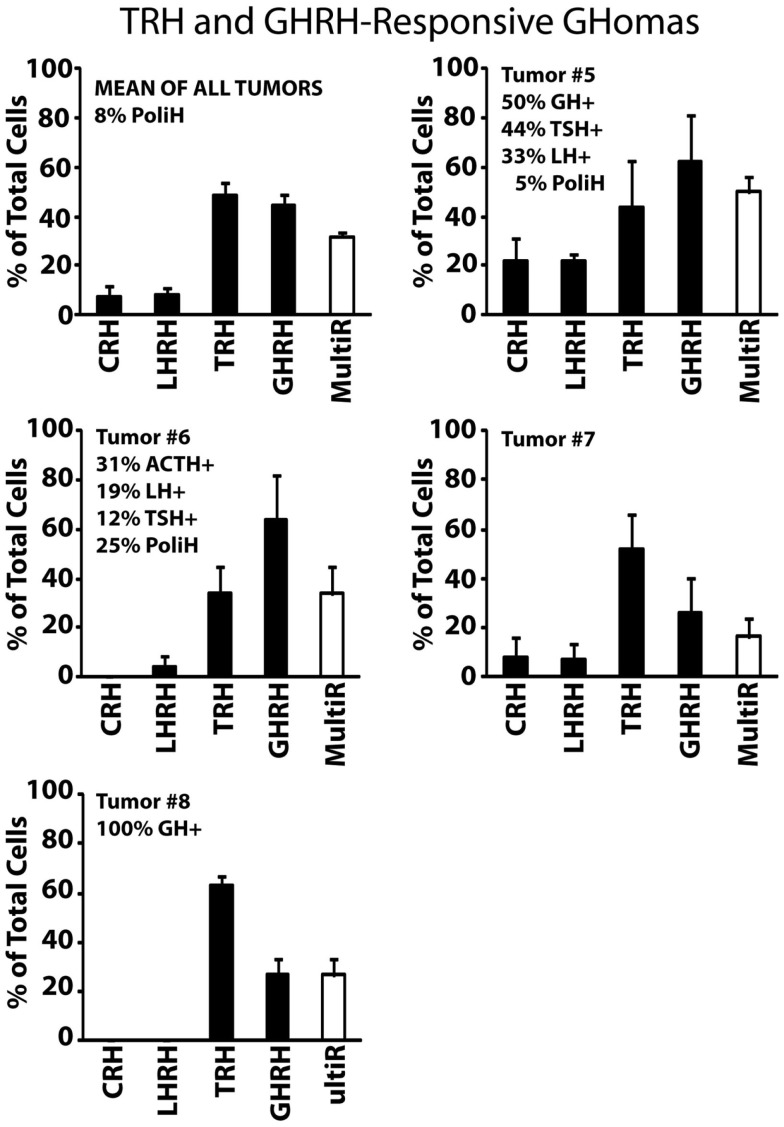
**Phenotypic characterization of TRH and GHRH-responsive GHomas**. Results from five individual tumors (#5 to #8) and the average of all five studied are shown. Data derived from 32 cells (two experiments, tumor #5), 23 cells (two experiments, tumor #6), 31 cells (four experiments, tumor #7), and 11 cells (two experiments, tumor #8). Average data are representative of 97 cells studied in 10 independent experiments. Other details as in Figure 2.

Type III, TRH and GHRH-responsive GHomas included most (five out of nine) GH-secreting adenomas analyzed in this study. These GHomas responded to both TRH and GHRH showing little or no response to other HRHs (Figure 3). Tumors #5 and #6 responded more prominently to GHRH (~60% of the cells) than to TRH (~30–40% of the cells) but tumors #7 and #8 responded more frequently to TRH (with 50–60%) than to GHRH. This group of tumors is the most heterogeneous one in terms of hormone content including tumors storing multiple AP hormones. Tumor #8 constitutes a clear example that may account for paradoxical secretion. This tumor is made of cells responding most frequently to TRH (>70%) but all cells store GH (100%). Immunofluorescence was not performed on cultured cells from tumor #7 owing to technical problems.

### Characterization of human non-functioning pituitary adenomas

We have characterized also individual cells derived from 11 human NFPAs. There was a large degree of functional differences among the NFPAs studied. According to hormone storage and responses to the HRHs, we have pooled also the NFPAs studied here in three different groups with distinct phenotypic characteristics: Type I, non-functioning ACTHomas; Type II, non-functioning gonadotropinomas, and Type III, non-functioning, null cell adenomas.

**Figure 4:**
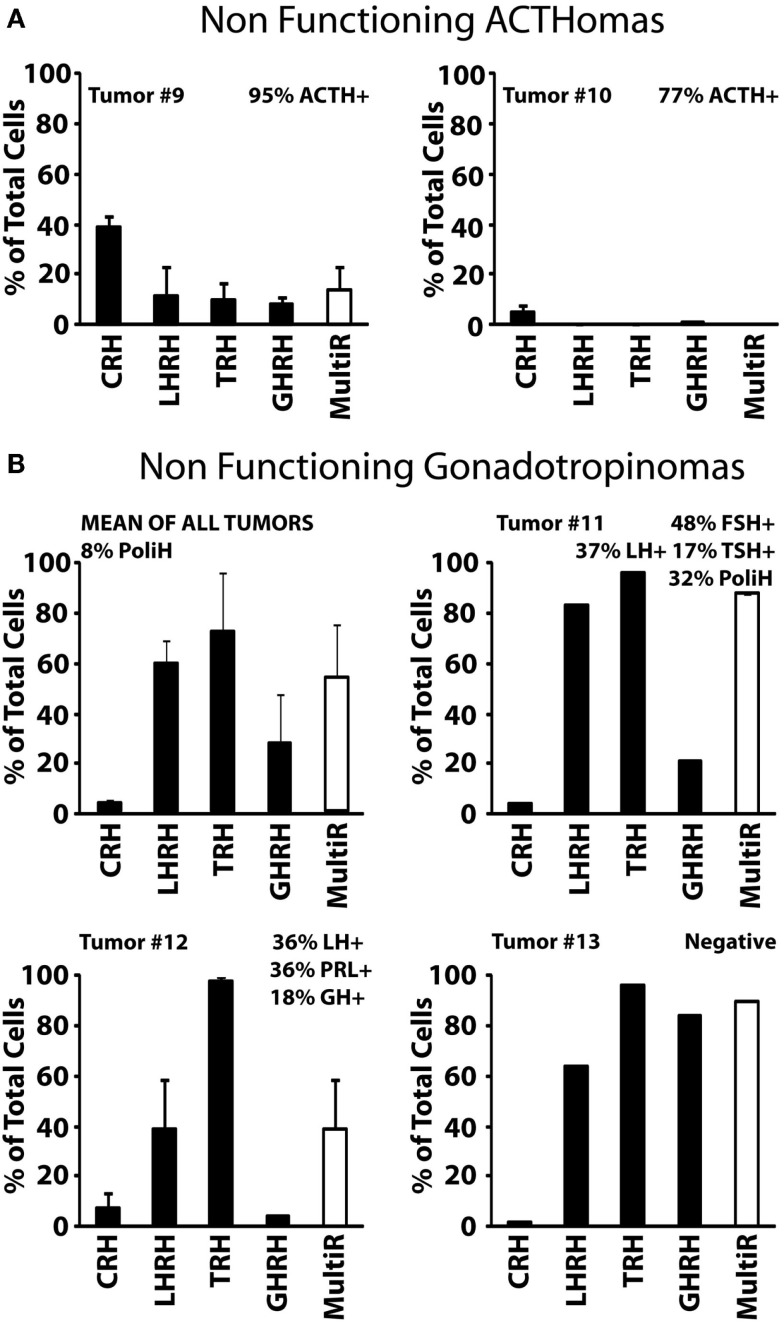
**Phenotypic characterization of non-functioning pituitary adenomas (NFPAs) with ACTH immunopositivity**. **(A)** Analyses of two NFPA are shown. Percents of cells responding to each HRH with a calcium rise larger than 50 nM are shown. The white bar at right shows the percentage of multiresponsive cells (those showing responses to >1 HRH). Data derived from 24 cells (2 experiments, tumor #9) and 188 cells (2 experiment, tumor #10). **(B)** Data from four tumors (#11, #12, and #13) and average are shown. Tumor #14 was made of cells responding (50%) to LHRH but not to any other HRH. About 25% of cells in this tumor stored either FSH or LH (not shown). Data derived from 139 cells (1 experiment, tumor #11), 38 cells (2 experiments, tumor #12), 50 cells (1 experiment, tumor #13), and 32 cells (2 experiments tumor #14). Average data are representative of 259 cells studied in 4 independent experiments. Other details as in Figure 2.

**Figure 5:**
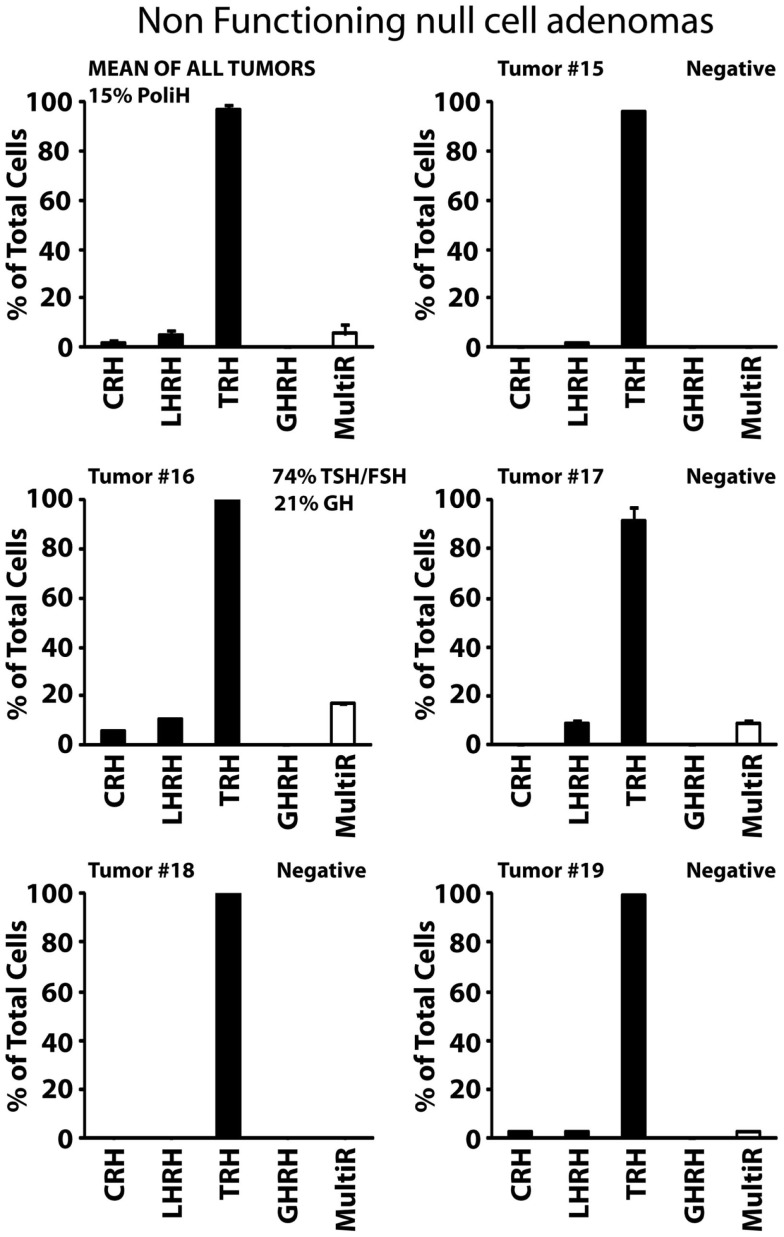
**Phenotypic characterization of non-functioning, null cell adenomas**. Results from five individual tumors (#15 to #19) and the average of all five studied are shown. Data derived from 54 cells (1 experiment, tumor #15), 19 cells (1 experiment, tumor #16), 22 cells (2 experiments, tumor #17), 27 cells (2 experiments, tumor #18), and 96 cells (2 experiments, tumor #19). Average data are representative of 218 cells studied in 8 independent experiments. Other details as in Figure 2.

Type I, non-functioning ACTHomas includes only two tumors (Tumors #9 and #10). Most cells in these two adenomas stored ACTH. In addition, cells were either partially (#9) or barely (#10) sensitive to CRH. Storage of other hormones or sensitivity to additional HRH receptors was not observed (Figure 4A). Type II, non-functioning gonadotropinomas (Tumors #11 to #14) includes four tumors. Most cells in this group showed Ca^2+^ responses to TRH and LHRH, and less frequently to GHRH. Responses to CRH were not observed (Figure 4B, Tumor #14 data not shown). Hormone content was variable among adenomas but the most frequent stored hormones were gonadotropins. Interestingly, gonadotropins were co-stored with additional AP hormones including TSH, PRL, GH, and even ACTH. The overall fraction of multiresponsive cells was as high as 70% of the cells. Finally, type III, non-functioning null cell adenomas included the most abundant group (5 out of 11) of NFPAs tested here. These adenomas showed a very homogeneous phenotype strikingly different from the previous ones. In the five tumors studied (#15 to #19), all the cells showed strong responses to TRH but not to any other HRH. Regarding hormone storage, all adenomas stored apparently no hormone except one that stored mostly TSH and FSH with a lower fraction of cells storing GH (Figure 5).

## Discussion

The pituitary gland is considered the master gland in charge of the endocrine system. Input from the hypothalamus in the form of hypothalamic releasing (and inhibiting) factors controls the release of specific AP hormones. AP hormones, in turn, modulate the activity of downstream peripheral glands and tissues that feedback at both hypothalamic and pituitary levels. Although pituitary tumors are generally benign and do not form metastasis, they have usually a strong impact on patient’s health due to chronic hormone imbalances and/or tumor mass effects. Pituitary tumors are typically characterized by its large heterogeneity. This is expected to some extent since the AP is made of five different cell types characterized by storage of different AP hormones and sensitivity to different hypothalamic releasing and inhibiting factors. However, classification of pituitary tumors is highly controversial. To improve tumor typing and gain insights on pituitary adenoma ontogeny, we have characterized hundreds of individual cells from a series of twenty human pituitary tumors classified clinically as GHomas and NFPAs, two categories that comprise nearly 40% of all pituitary tumors. The single-cell phenotypes of other frequent pituitary adenomas including prolactinomas, adenomas related to MEN-1 and Cushing, and a few NFPAs has been reported previously (17).

We found that most GHomas were made of polyhormonal cells storing GH and showing calcium responses to both TRH and GHRH (type III GHomas). Other GHomas stored only GH but responded to all four HRH (type II GHomas) or were polyhormonal cells lacking responses to any HRH (type I GHomas). The presence of multifunctional cells (multiresponsive and/or polyhormonal cells) in human GHomas may explain, in a simple way, the occurrence of paradoxical secretion, which has been frequently reported in pituitary tumors (6–13). Consistently, the most common phenotype of GHomas made of polyhormonal GH cells and showing responses to both TRH and GHRH may explain paradoxical secretion of GH induced by several HRHs, most notably TRH. In addition, it may also explain paradoxical secretion of other AP hormones by GHomas. No information is available on single-cell phenotypes in the human, normal AP. Several attempts have been carried out in samples from organ donors but cell cultures have failed so far in our hands. However, normal GH cells from mice are mostly monohormonal cells responding only to GHRH (18, 25) and the multifunctional phenotype seem to show up only in demanding physiological situations, a process that has been related to pituitary plasticity. Accordingly, the abundance of multifunctional phenotypes in human GHomas suggests that adenomas may arise as a consequence of dysfunctional pituitary plasticity as suggested previously for other pituitary adenomas (17).

Regarding NFPAS, we found that a large fraction of NFPAs studied were made of cells holding a very particular phenotype: These cells are rather homogenous, store no detectable hormone (null cells) and responded only to TRH (type III non-functioning adenomas). Other NFPAs contained polyhormonal gonadotropes responding to LHRH and TRH (type II non-functioning adenomas) or monohormonal corticotropes lacking responses to HRHs (type I non-functioning adenomas). These results are consistent with a previous report showing that a fraction of NFPA patients show abnormal LH secretion in response to TRH (26). Accordingly, our type III and type II NFPAs may well correspond to silent gonadotrope adenomas expressing TRH receptors and differing simply in the level of expression of gonadotropins and LHRH receptors. We had previously reported a similar analysis in a small number of NFPAs (17). One of these previously reported adenomas was made of cells storing both GH and PRL reflecting probably a silent somatoprolactinoma. The other two previously reported adenomas were made of cells storing no hormone or mostly ACTH and showing responses to CRH and TRH and to a lesser extent to GHRH suggesting a corticotrope ontogeny. Accordingly, the two previously reported NFPAs resemble the type 1 NFPA reported here.

Hypothalamic releasing hormones are considered more than simple secretagogues as they are also able to promote proliferation of target cells. For example, it has been reported that excessive secretion of GHRH or overexpression of GHRH receptors may result in deregulated proliferation of somatotrophs, leading to hyperplasia, and neoplastic transformation (27). Interestingly, a large fraction of GHomas and most NFPAs studied responded largely to TRH either alone or in combination with other HRHs. If confirmed, these results suggest that TRH antagonists could be considered for the treatment of a large fraction of NFPAs. This could be particularly interesting for type III NFPAs accounting for about 50% of all NFPAs studied here as they are responsive only to TRH but not to any other HRHS. According to our results, this possible treatment could be less effective for type II NFPAs as they are responsive also to LHRH and GHRH. No effect of TRH antagonists is expected in type I NFPAs as they lack calcium responses to TRH. However, in this particular case, a possible effect of CRH antagonists is expected as cells from these tumors respond to CRH but not to any other HRH. Further research is required to confirm these results and to validate TRH and CRH receptors as targets for NFPAS.

Finally, it has been previously suggested that cell phenotypes within the normal AP are not static (25, 28). Instead, they are moving targets that may undergo dramatic changes in demanding physiological situations such as maturation, puberty, and senescence (29). It is worth noting that even a short period of stress may lead to significant changes in AP cell composition, particularly in females (30). One interesting possibility is that this plasticity, a particular characteristic of AP cells, may remain in the tumor and be activated by extrinsic factors, thus providing an explanation for changes in cell phenotypes in tumor recurrences. This plasticity may also contribute to explain phenotypic differences among cells within the same tumor. Further research is required to understand phenotypic heterogeneity and plasticity in the AP and pituitary adenomas.

## Conflict of Interest Statement

The authors declare that the research was conducted in the absence of any commercial or financial relationships that could be construed as a potential conflict of interest.
